# Comparison of the prevalence and associated factors of chronic kidney disease diagnosed by serum creatinine or cystatin C among young people living with HIV in Uganda

**DOI:** 10.1186/s12882-024-03865-8

**Published:** 2024-11-25

**Authors:** Esther M. Nasuuna, Laurie A. Tomlinson, Robert Kalyesubula, Chido Dziva Chikwari, Barbara Castelnuovo, Yukari C. Manabe, Damalie Nakanjako, Helen A. Weiss

**Affiliations:** 1 Non-communicable Diseases Program, Medical Research Council/Uganda Virus Research Institute and London School of Hygiene and Tropical Medicine Uganda Research Unit, Entebbe, Uganda; 2 Infectious Diseases Institute, Makerere University, College of Health Sciences, Kampala, Uganda; 3 Department of non-Communicable Disease Epidemiology, London School of Hygiene and Tropical Medicine, Keppel Street, London, WC1E 7HT UK; 4 Departments of Physiology and Medicine, Makerere University College of Health Sciences, Kampala, Uganda; 5 Biomedical Research and Training Institute, Harare, Zimbabwe; 6 MRC International Statistics and Epidemiology Group, London School of Hygiene & Tropical Medicine, London, UK; 7 Department of Medicine, Johns Hopkins University School of Medicine, Baltimore, MD USA; 8 Department of Medicine, School of Medicine, College of Health Sciences, Makerere University, Kampala, Uganda

**Keywords:** Prevalence, Chronic kidney disease, Young people, HIV, Africa, HIV comorbidities

## Abstract

**Introduction:**

Young people living with HIV (YPLHIV) are at increased risk of developing chronic kidney disease (CKD) which is associated with high mortality and morbidity. Early diagnosis is important to halt progression. We aimed to estimate the prevalence and factors associated with CKD among YPLHIV in Kampala, Uganda, and to compare serum creatinine and cystatin C for early diagnosis of CKD in this population.

**Methods:**

A cross-sectional study with YPLHIV aged 10 to 24 years was conducted in seven HIV clinics. Participants provided a urine and blood sample to measure urinary albumin, proteinuria, serum creatinine and cystatin C levels at baseline and after three months. The estimated glomerular filtration rate (eGFR) was calculated using CKDEPI 2021, Cockroft-Gault and bedside Schwartz equations using creatinine or cystatin C. The albumin creatinine ratio (ACR) and proteinuria were measured. CKD was defined as either eGFR < 60 ml/min/1.73m^2^ or < 90 ml/min/1.73m^2^ or ACR above 30 mg/g on two separate occasions. Univariable and multivariable logistic regression were used to estimate adjusted odds ratios (aOR) and 95% confidence intervals (CI) for factors associated with CKD.

**Results:**

A total of 500 participants were enrolled. Most were female (56%; *n* = 280) and aged 10 to 17 years (66.9%; *n* = 335). CKD prevalence ranged from 0 to 23% depending on the criteria, equation and biomarker used. Cystatin C-based equations estimated higher prevalence of CKD compared to creatinine-based ones. Prevalence of ACR above 30 mg/g was 10.1% and of proteinuria 29%. Factors independently associated with CKD were age (aOR = 1.42; 95% CI:1.30–1.51) and male sex (aOR = 3.02; 95% CI:1.68–5.43).

**Conclusion:**

CKD prevalence among YPLHIV varied substantially depending on definitions used and the current definition would likely lead to missed cases of CKD among YPLHIV. Estimating equations should be validated against measured GFR in YPLHIV and the optimal definition of CKD in this vulnerable population should be revised to optimise detection and opportunities for reducing disease progression.

**Clinical trial number:**

Not applicable.

**Supplementary Information:**

The online version contains supplementary material available at 10.1186/s12882-024-03865-8.

## Introduction

Prevalence of chronic kidney disease (CKD) is increasing globally [1]. CKD is defined as abnormalities in kidney structure or function present for three or more months [2]. The Global Burden of Disease study estimates CKD prevalence at 9.1% (95% CI 8.5%-9.8%) with geographic variation [3]. Studies in Sub-Saharan Africa (SSA) have found prevalence ranging from 6 to 48% depending on the population, the definitions used, and the measurements taken [4–6].

CKD is associated with high morbidity and mortality as diagnosis is usually delayed, often occurring after kidney failure due to its insidious onset [7]. Kidney failure can only be treated with expensive kidney replacement therapies (KRT) that are not readily available in low and middle-income countries [8]. Early diagnosis before marked loss of kidney function and severe damage is important to minimise risk of progression to kidney failure and cardiovascular events [9–11].

Diagnosis of CKD is based on the level of glomerular filtration rate (GFR) and markers of kidney damage such as protein excretion into the urine shown by proteinuria or albuminuria [12]. GFR can either be measured directly (mGFR) or estimated (eGFR) with a specific biomarker and one of the estimating equations [13]. Most commonly, serum creatinine and cystatin C estimating equations are used to estimate GFR [14]. Serum creatinine is widely available and relatively cheap [15] but has limitations as it is influenced by muscle mass, physical activity and general health status [16] as well as high analytic variability [15]. Cystatin C is not affected by these conditions as it is produced by most nucleated cells and has uniform generation despite individual differences in people and situations [16–18]. However, it is affected by conditions of high inflammation, corticosteroid use and thyroid disease [16, 19]. Cystatin C more accurately estimated measured GFR compared to creatinine in a large cohort study done across Uganda, Malawi and South Africa that recommended the use of Cystatin C in African populations [4].

Young people living with HIV (YPLHIV) are at higher risk of CKD than young people not living with HIV [20]. CKD risk is associated with high HIV viremia (> 4000 copies per ml), severe immunosuppression (CD4 cell count < 200 cells/ml), infection with hepatitis C virus, diabetes mellitus (DM), hypertension, use of drugs that treat opportunistic infections, and toxicity due to anti-retroviral therapy (ART) from tenofovir disoproxil fumarate (TDF) and indinavir [6, 21–24]. Further, YPLHIV in SSA are particularly vulnerable to developing CKD compared to adults living with HIV due to late HIV diagnosis and initiation on ART, poorer adherence to ART complicated by high viremia and low CD4 cell counts [25–27].

Although YPLHIV are at high risk of CKD, little is known about CKD prevalence, the best biomarker to diagnose CKD and factors associated with CKD in this vulnerable group. Therefore, we sought to study this among YPLHIV in Kampala, Uganda.

## Methods

### Study design and setting

This cross-sectional study was conducted in the HIV clinics of seven urban public health facilities from the 12th of April 2023 to 31st January 2024 in Kampala, Uganda. These offer comprehensive HIV care to children (aged below 18 years) and adults (aged 18 years and above).

### Study population and sampling

The study included YPLHIV aged 10–24 years with presumed perinatal HIV infection (defined as being diagnosed with HIV before 10 years of age with self-report of no sexual debut or blood transfusion prior to diagnosis). Pregnant YPLHIV were excluded. Systematic random sampling was used to identify potential participants from all YPLHIV enrolled in the seven HIV clinics from electronic medical records. They were ordered by age at diagnosis and every third person invited to join the study. A sample size of 500 was powered to detect a prevalence of CKD between 16% and 24%.

### Study procedures

Eligible participants were invited to the HIV clinic through a phone call where they were screened, consented, and enrolled. A trained study team member conducted an interview with the participant and completed a questionnaire to record demographic information, symptoms, risk factors and the relevant medical history such as prior diagnosis with diabetes mellitus (DM) or hypertension. Anthropometric measurements (mid-upper arm circumference (MUAC), weight and height) were taken. Weight was assessed using a digital weighing scale, height using a stadiometer and blood pressure (BP) using a digital BP machine with a paediatric cuff for younger participants. Body composition monitoring was conducted using bioimpedance impedance spectroscopy (BIS) to measure body fat, muscle mass and visceral fat. Participants provided a spot urine sample (20 mls) as well as 8 ml of venous blood. Urine dipstick was done at the facility to determine proteinuria and other urinary abnormalities. The samples were stored in a cooler box before transfer to the study laboratory on the same day. Participants that had kidney abnormalities (eGFR < 90 ml/min/1.73m2 or ACR > = 30 mg/g) at the initial visit were invited back for another urine and blood test at three months to confirm CKD.

### Laboratory methods and testing

In the laboratory, serum creatinine, urinary albumin and cystatin C levels were determined. Those with an albumin creatinine ratio (ACR) > = 30 mg/g or eGFR < 60 ml/min/1.73m^2^ at baseline were followed-up after three months to confirm the KDIGO guideline-recommended clinical diagnosis of CKD. Cystatin C was measured by particle-enhanced immunoturbidimetric assay on Roche Cobas C311 platform with Tina-quant Cystatin C Gen.2. Creatinine was measured using the enzymatic calorimetric method using an isotope dilution mass spectroscopy (IDMS) traceable standard reference material on the Cobas Integra 400 plus machine with Creatinine Plus Version 2 (CREP2), Roche Diagnostics. The urine albumin was quantified using the immunoturbidimetric assay on the Roche Cobas C311 platform using Tina-quant Albumin Gen2, (Roche Diagnostics). Prior to testing, the machines were calibrated according to manufacturer instructions. Urinalysis by dipstick was done with AYDMED urinalysis Reagent Test Strips (Sungo Europe B.V Amsterdam) to determine presence of urobilinogen, bilirubin, ketones, blood, proteins, nitrites, leucocytes, glucose, specific gravity, pH, and ascorbic acid [28].

**Diagnosis of CKD** was based on the kidney disease improving global outcomes (KDIGO) guidelines and staging [29], i.e. (1) markers of kidney damage such as an albumin: creatinine ratio > = 30 mg/g, or (2) eGFR < 60 ml/min/1.73m^2^, with these abnormalities confirmed with a repeat test after three months [30]. Since a normal GFR is between 90 and 120 ml/min/1.73m^2^, we also considered an eGFR cut-off below 90 ml/min/1.73m^2^ which is regarded as stage 2 CKD as abnormal in such a young population [31]. The initial baseline measurement of either eGFR below 90 ml/min/1.73m2 or ACR above 30 mg/g was regarded as kidney impairment as CKD status was not yet established at that point.

To explore CKD definition in this cohort that included children and where chronic disease had affected pubertal development and mean body and muscle mass, we primarily used a range of GFR estimating equations and eGFR cut offs that reflected contemporary practice for adults and children and/or sought to adjust for body size. eGFRscr was estimated using the following creatinine-based equations: CKD Epidemiology collaboration (CKDEPI) 2021 [14] which is the current KDIGO recommended equation for GFR estimation in adults, the Bedside Schwartz [32] which is KDIGO recommended for children, and Cockroft-Gault because it takes into consideration stature by including height. eGFRcystc was estimated using the following cystatin C-based equations: Schwartz cystatin C [33] and CKDEPI 2012 [34]. For completeness prevalence was also estimated using other relevant equations such as Full Age Spectrum [35], CKDEPI40 [36] and Pierce U25 [37] among others, and in combination with ACR and proteinuria.

### Data management and statistical analysis

Data were collected in REDCap and analysed with STATA statistical software version 18 (STATA Corp USA). Demographic data were collected on age, sex, address, religion, tribe, assets one owned and marital status. Symptoms such as loss of weight, anorexia, nausea, vomiting, facial puffiness in the morning and urinary volume abnormalities were collected. Risk factors such as prior diagnosis with hypertension, diabetes mellitus, tuberculosis, prolonged use of non-steroidal anti-inflammatory drugs, type of anti-retroviral drugs used, duration living with HIV and viral suppression were collected. Viral suppression was considered as an HIV viral load below 1000 copies/ml. Hypertension was classified according to the American Academy of Paediatrics (AAP) guidelines as being above the 95th percentile for age and sex below 13 years and above 130/80mmhg in those above 13 years [38]. Muscle mass was abnormal if below 33.3 for males and 24.3 for females. Social economic status was divided into three using principal component analysis. Demographic data were summarised in percentages or means (standard deviation) and median (interquartile range). We determined the eGFR at the initial visit and at the three month follow up visit. The distribution of eGFRs estimated with different equations was shown in a Kernel density plot. CKD prevalence diagnosed by either creatinine or cystatin C was calculated. Univariable logistic regression was used to estimate odds ratios (OR) of factors associated with CKD for each of the five equations used, respectively. All variables with *p* < 0.2 in the univariable model, and a-priori identified variables known to be associated with CKD (age, sex, HIV viral suppression, blood pressure) were then included in a multivariable logistic regression model for each of the five equations. We assessed for multicollinearity to ensure the accuracy of the estimates, between age, blood pressure, body mass index, mid upper arm circumference using the variance inflation factor. If the factors were below 5 and the tolerance not close to 0, multicollinearity was not present [39].

### Ethical considerations

Ethical approval was received from the Uganda Virus Research Institute (UVRI) Research Ethics Committee (reference number GC/127/946), the Uganda National Council of Science and Technology (HS2578ES) and the London School of Hygiene and Tropical Medicine institutional review board (28797). Information about the study appropriate for adults, semi-literate adults and children was provided in an information booklet that was read to the participants and caregivers. All the participants more than 18 years of age provided a written informed consent. Those below 18 years of age provided assent, and their caregivers provided written informed consent. If a child refused to provide assent even after their caregiver had provided consent, that child was not enrolled into the study. All participants had the option to withdraw at any point during the research. All participants with suspected CKD were referred to a nephrologist for management.

## Results

Of 532 YPLHIV invited to participate, 500 were enrolled as 32 declined to participate (Table 1). The majority were female (56.0%; *n* = 280), children aged 10–17 years (66.9%; *n* = 335) and living in Kampala (58.9%; *n* = 295). Females had better nutritional indicators than males - they were less likely to be underweight (26.4% vs. 48.9%; *p* < 0.001), not stunted (85.6% vs. 76.9%; *p* = 0.03), and to have normal mid upper circumference (92.9% vs. 87.7%; *p* = 0.05). No participant had a prior diagnosis of DM or hypertension.

**Table 1:** Demographic and clinical characteristics of the study participants by sex

	Male	Female	Total
	*N* = 220 (44%)	*N* = 280 (56%)	*N* = 500
Age in years (mean, SD)	16.5 (3.8)	16.3 (3.5)	16.4 (3.6)
Age category			
Children	143 (65.0)	191 (68.2)	334 (66.8)
Adults	77 (35.0)	89 (31.8)	166 (33.2)
Address			
Kampala	123 (55.9)	171 (61.1)	294 (58.8)
Wakiso	80 (36.4)	99 (35.4)	179 (35.8)
Other districts	17 (7.7)	10 (3.6)	27 (5.4)
Religion			
Christian	158 (71.8)	204 (72.9)	362 (72.4)
Moslem	62 (28.2)	73 (26.1)	135 (27.0)
Other	0 (0.0)	3 (1.1)	3 (0.6)
Social Economic Status			
Lowest	78 (35.5)	100 (35.7)	178 (35.6)
Middle	61 (27.7)	94 (33.6)	155 (31.00
Highest	81 (36.8)	86 (30.7)	167 (33.4)
School going			
No	58 (26.4)	67 (23.9)	125 (25.0)
Yes	162 (73.6)	213 (76.1)	375 (75.0)
Marital Status			
Married	3 (1.4)	16 (5.7)	19 (3.80)
Never married	217 (98.6)	264 (94.3)	481 (96.2)
Tribe			
Ganda	149 (67.7)	174 (62.1)	323 (64.6)
Other tribes	65 (29.5)	86 (30.7)	151 (30.2)
Non-Ugandan	6 (2.7)	20 (7.1)	26 (5.2)
Weight in Kg mean (SD)	48.0 (12.4)	49.4 (12.4)	48.8 (12.4)
Body Mass Index^1^			
Normal	173 (79.0)	214 (76.4)	387 (77.6)
Underweight (< 18.5 kg/m^2^)	38 (17.3)	24 (8.6)	62 (12.4)
Overweight (> 25 kg/m^2)^	8 (3.7)	42 (15.0)	50 (10.0)
Stunting**			
Not stunted	123 (76.9)	184 (85.6)	307 (81.9)
Stunted	37 (23.1)	31 (14.4)	68 (18.3)
Mid Upper Arm Circumference ^2^			
Normal	192 (87.7)	260 (92.9)	452 (90.6)
Malnourished	27 (12.3)	20 (7.1)	47 (9.4)
Blood pressure^3^			
Normal	137 (62.8)	215 (77.1)	352 (70.8)
Elevated	39 (17.9)	27 (9.7)	66 (13.3)
Hypertensive	42 (19.3)	37 (13.3)	79 (15.9)
On TDF regimen			
No	61 (27.7)	66 (23.6)	127 (25.4)
Yes	159 (72.3)	214 (76.4)	373 (74.6)
On Dolutegravir			
Yes	210 (95.5)	273 (97.5)	483 (96.6)
No	10 (4.5)	7 (2.5)	17 (3.4)
Virally suppressed			
Yes	195 (89.5)	247 (88.5)	442 (88.9)
No	23 (10.5)	32 (11.5)	55 (11.1)
Muscle mass ^2^			
Normal muscle mass	164 (82.0)	235 (87.7)	399 (85.3)
Abnormal muscle mass	36 (18.0)	33 (12.3)	69 (14.7)

* Living outside Kampala/Wakiso region. # included those with no religion and those of African traditional religion. **Only those aged less than 19 years. 1 one missing, 2. 32 missing as their measurements were below threshold of the BIS machine.3 three missing a blood pressure reading KG-Kilogram, *N*-number, SD-standard deviation, TDF Tenofovir disoproxil fumarate

### Comparison of serum creatinine and cystatin C

**Fig. 1:**
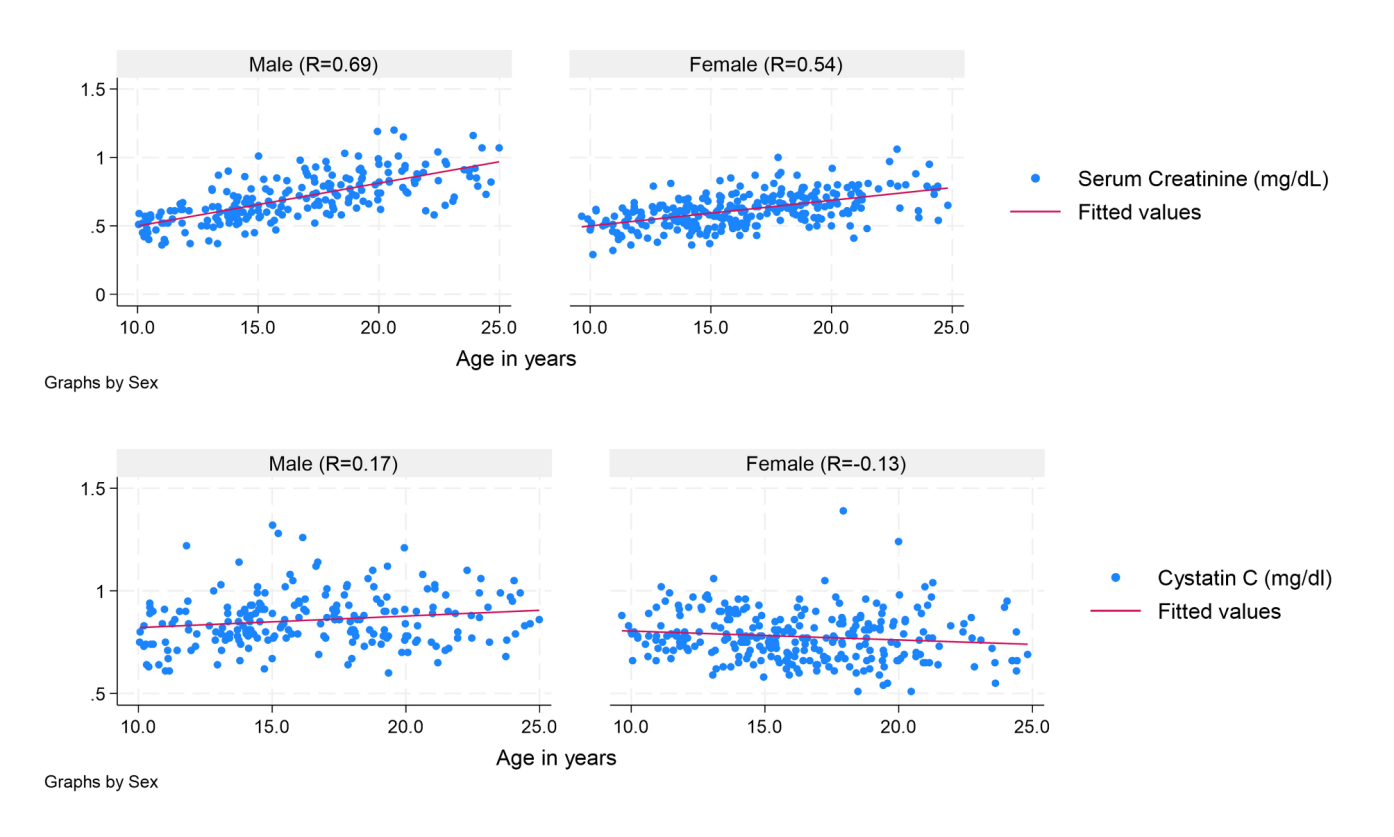
Relationship between serum creatinine and cystatin C and age for males and females Sample size was 494

The mean serum creatinine (scr) was 0.63 mg/dl (SD 0.15) with a range of 0.29 to 1.2 mg/dl. The mean scr was significantly different according to sex, age, presence of stunting or viral suppression. The mean cystatin C was 0.81 mg/dl (SD 0.13) with a range of 0.51 to 1.39 mg/dl. The mean cystatin C was higher in males at 0.86 mg/dl versus 0.78 mg/dl in females but with no other differences (Supplemental Table 1). Serum creatinine but not cystatin C was correlated with age and sex (Fig. 1).

### Distribution of the eGFR

**Fig. 2:**
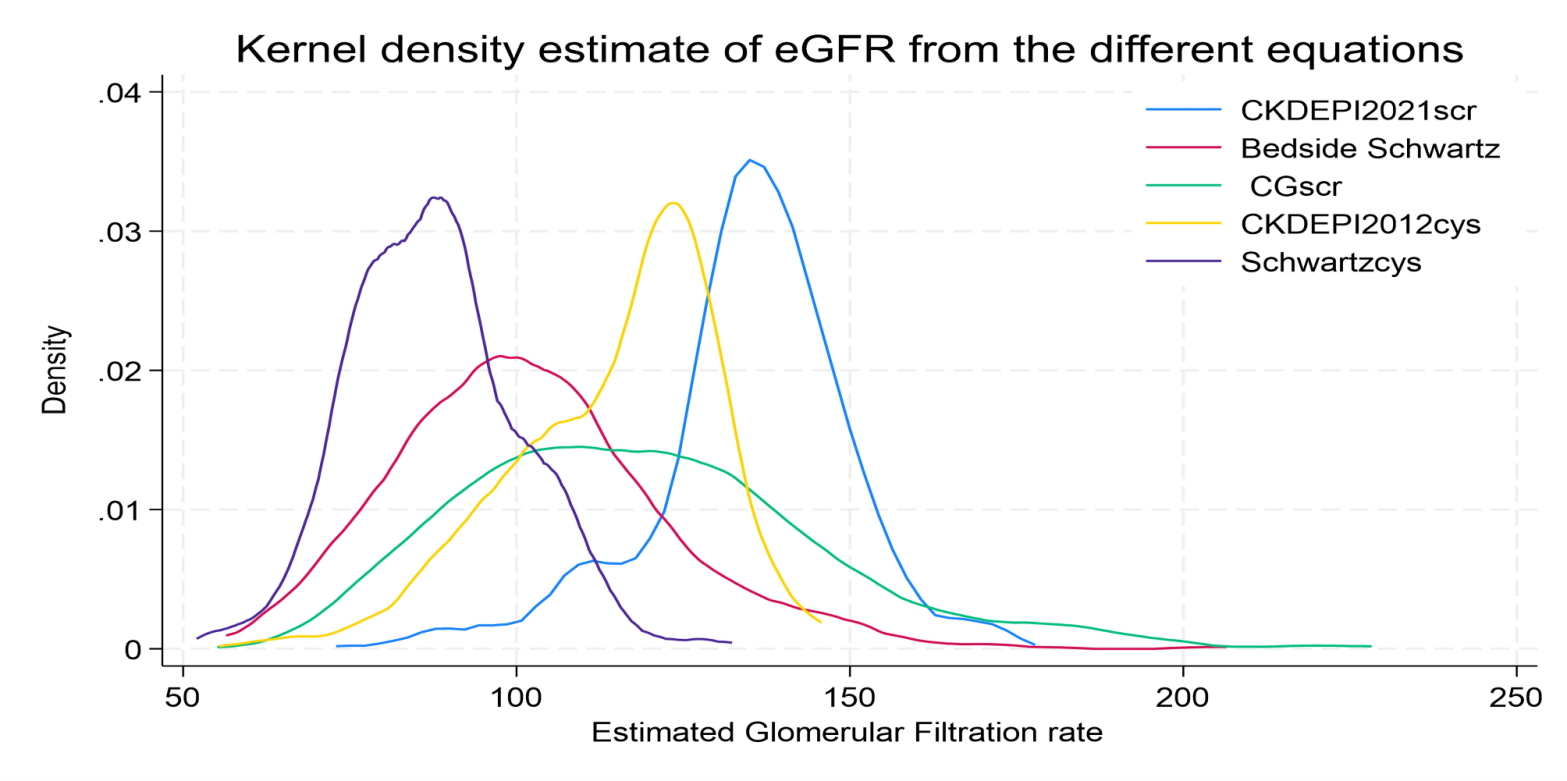
Kernel density plot showing the distribution of the eGFR according to different estimating equations and biomarkers

CKDEPI consistently gave higher eGFR readings for both creatinine and cystatin C, and the Schwartz cystatin C equation gave the lowest eGFR values (Fig. 2).

Scr serum creatinine, CG Cockroft Gault, cys cystatin C

### Prevalence of CKD and kidney impairment using eGFR

CKD and kidney impairment prevalence varied according to the eGFR cut-off, and the biomarker used. Using an eGFR < 60 ml/min/1.73m^2^ cut-off, the highest prevalence was with the Schwartz cystatin C equation which showed a 1.4%; 95% CI: 0.5–2.9% prevalence of kidney impairment and a 0.8%; 95% CI: 0.2–2.1% prevalence of CKD at the 3-month follow-up. The lowest prevalence was with the CKDEPI 2021 equation with a 0%; 95% CI 0-0.07% prevalence of both kidney impairment and CKD.

**Fig. 3:**
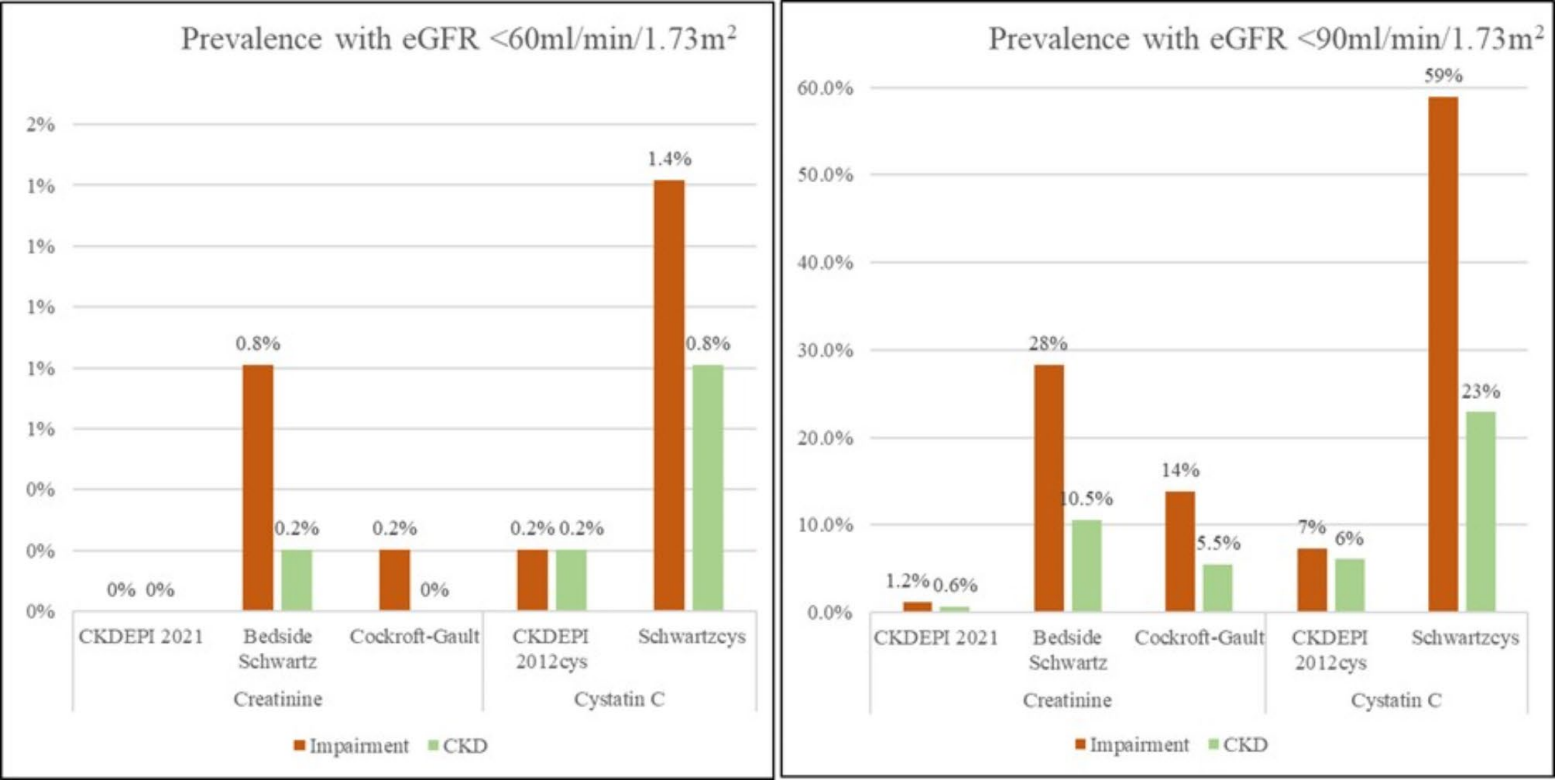
Prevalence of CKD according to the different estimating equations and biomarkers Sample size was 494

Similarly, using eGFR < 90 ml/min/1.73m^2^ cut-off, the highest prevalence was with the Schwartz cystatin C equation that showed a 58.9% (95% CI: 54.4–63.3%) prevalence of kidney impairment and a 23% (95% CI 19.2–26.8%) prevalence of CKD. The lowest with CKDEPI with a kidney impairment prevalence of 1.2% (95% CI 0.5–2.6%) and a 0.6%; 95% CI: 0.01–1.7% prevalence of CKD (Fig. 3). Prevalence using other eGFR equations ranged from 0 to 27.5% (Supplementary Table 2).

### Prevalence of CKD according to eGFR and ACR

All participants were staged according to combined baseline eGFR using cystatin C and ACR to assess risk of progression [29]. Overall, 436 (88.3%) participants had low risk of progression ^1^, 53 (10.7%) had intermediate risk of progression ^2^ and 5 (1.0%) were at high risk of progression ^3^ with no one in the highest risk category ^4^. (Table 2).

**Table 2:** All participants’ CKD status staged according to estimated GFR from cystatin C and albumin creatinine ratio at baseline

eGFR and ACR categories		ACR categories in mg/g			
		< 30 Normal to mildly increased	30–300 Moderately increased	> 300 Severely increased	Total numbers
eGFR in ml/min/1.73m^2^	Stage	A1	A2	A3	
>=90 Normal and high	G1	178 (36.0%)^1^	24 (4.9%)^2^	1 (0.2%)^3^	203 (41.1%)
60–89 Mild reduction	G2	258 (52.2%)^1^	24 (4.9%)^2^	2 (0.2%)^3^	284 (57.5%)
45–59 Mild to moderate reduction	G3a	5 (1.0%)^2^	2 (0.4%)^3^	0 (0%)^4^	7 (1.4%)
		441 (89.3%)	50 (10.1%)	3 (0.6%)	494* (100%)

eGFR estimated glomerular filtration rate, ACR Albumin creatinine ratio, *6 participants were missing serum creatinine and cystatin C results. 1 low risk of progression, 2 intermediate risk of progression, 3 high risk of progression, 4 highest risk of progression

### Prevalence of CKD according to markers of kidney damage

Urinalysis showed that 143 (29%) participants had proteinuria on dipstick. Prevalence of proteinuria was similar for those with eGFR > = 90 ml/min/1.73m^2^ and < 90 ml/min/1.73m^2^ (24.6% vs. 31.7% *p* = 0.17). At baseline, 10.1% [50] and at follow up, 3.8% [19] participants had an ACR > = 30 mg/g.

### Factors associated with CKD

Factors associated with CKD varied with the equation and biomarker used for those with an eGFR < 90 ml/min/1.73m^2^ (Table 3) but was largely associated with male sex (with the exception of CKDEPI2021), viral non-suppression (by the cystatin C based equations), increasing age (by the CKDEPI and Bedside Schwartz equations), and being overweight (with the exception of the Cockroft-Gault equation). CKD was also associated with proteinuria (by the CKDEPI 2012 equation) and being on a TDF-based regimen (by the Bedside Schwartz equation). There was no evidence that CKD was associated with high blood pressure, muscle mass, and ACR.

**Table 3:** Factors associated with having CKD (eGFR < 90 ml/min/1.73m^2^) among study participants according to the different estimating equations and biomarkers

	eGFR < 90 ml/min/1.73m2 from Cystatin C based Equations				eGFR < 90 ml/min/1.73m^2^from Serum Creatinine Based Equations					
	CKD EPI 2012 (*n* = 36/494)		Schwartz Cystatin (*n* = 291/494)		CKDEPI 2021 (*n* = 6/494)		Bedside Schwartz (*n* = 140/494)		Cockroft Gault (*n* = 68/493)	
	Unadjusted OR	Adjusted OR*	Unadjusted OR	Adjusted OR	Unadjusted OR	Adjusted OR	Unadjusted OR	Adjusted OR	Unadjusted OR	Adjusted OR
Age in years	1.14 (1.03–1.25)	1.13 (1.01–1.27)	0.98 (0.94–1.04)	0.99 (0.94–1.04)	1.50 (1.13–1.98)	1.45 (1.01–2.14)	1.40 (1.30–1.51)	1.42 (1.28–1.58)	0.99 (0.94–1.04)	1.08 (0.96–1.22)
Age categorized										
> 18	1	1	1		1		1		1	
< 18	0.47 (0.33–0.92)		1.06 (0.73–1.55)		0.09 (0.01–0.83)		0.17 (0.11–0.26)		0.89 (0.52–1.52)	0.63 (0.23–1.77)
Sex										
Females	1		1		1		1		1	
Males	2.42 (1.2–4.92)	2.84 (1.27–6.31)	3.21 (2.18–4.71)	3.13 (2.12–4.65)	0.64 (0.12–3.52)	0.75 (0.11–4.92)	2.22 (1.49–3.32)	3.02 (1.68–5.43)	3.21 (2.18–4.71)	0.32 (0.16–0.60)
Blood pressure										
Normal	1	1	1		1	1	1	1	1	1
Elevated	0.59 (0.17–1.98)	0.41 (0.86–1.90)	1.69 (0.95–2.98)	1.42 (0.79–2.59)	0.07 (0.00-0.63)	0.35 (.-.)	1.19 (0.66–2.17)	1.02 (0.45–2.31)	0.41 (0.21–0.78)	0.26 (0.08–0.80)
Hypertensive	0.98 (0.39–2.45)	0.69 (0.26–1.89)	1.25 (0.76–2.06)	1.14 (0.67–1.94)	0.45 (0.04–4.04)	1.61 (0.24,10.63)	2.42 (1.46–4.02)	1.69 (0.89–3.29)	0.73 (0.35–1.50)	0.58 (0.18–1.86)
Viral suppression										
Suppressed	1	1	1	1	1		1		1	
Non suppressed	3.11 (1.38–7.04)	3.27 (1.29–8.32)	2.09 (1.11–3.97)	2.29 (1.18–4.44)	1.01 (0.00-7.12)	0.92 (.,.)	0.42 (0.19–0.91)	0.34 (0.13–0.89)	0.63 (0.24–1.64)	0.61 (0.21–1.74)
CD4 T cell count at baseline										
> 500	1	1	1		1		1		1	
200–500	2.3 (0.98–5.38)	1.41 (0.55–3.59)	1.54 (0.96–2.48)		1.52 (0.03–29.58)		1.86 (1.03–3.04)	0.66 (0.35–1.25)	1.08 (0.56–2.10)	
< 200	3.84 (1.5–9.38)	2.59 (0.97–6.92)	1.76 (0.96–3.22)		5.34 (0.38–75.78)		1.89 (1.04–3.46)	0.65 (0.29–1.44)	1.22 (0.56–2.68)	
Social Economic Status										
Least	1		1		1		1		1	
Middle	1.03 (0.42–2.49)		0.96 (0.62–1.49)		2.28 (0.81–2.21)		1.34 (0.62–1.49)	1.32 (0.69–2.51)	1.04 (0.56–1.92)	
Highest	1.47 (0.66–3.31)		1.15 (0.74–1.78)		1.85 (1.15–2.99)		1.15 (0.75–1.78)	1.40 (0.75–2.64)	0.81 (0.43–1.52)	
Body Mass Index										
Normal	1		1		1		1		1	1
Underweight	1.69 (0.82–3.43)		1.36 (0.92–2.01)		0.29 (0.00-2.30)		0.31 (0.18–0.51)	0.74 (0.32–1.68)	4.03 (2.29–7.09)	7.23 (3.33–15.7)
Overweight	1.27 (0.35–4.56)		1.24 (0.63–2.43)		3.11 (0.27–22.51)		1.81 (0.94–3.51)	1.98 (0.68–5.74)	0.19 (0.00-1.12)	
Weight in Kg	1.02 (0.99–1.04)		1.01 (0.99–1.02)		-		1.06 (1.04–1.09)	0.99 (0.96–1.04)		
Stunting										
Not stunted	1		1		1		1		1	
Stunted	1.42 (0.50–4.02)		0.88 (0.52–1.51)		-		0.67 (0.31–1.43)		1.81 (0.87–3.73)	
Mid Upper Arm Circumference										
Normal	1		1		1		1		1	
Malnourished	1.64 (0.60–4.44)		0.74 (0.40–1.36)		-		0.43 (0.18–0.98)	0.82 (0.29–2.39)	4.05 (2.07–7.93)	1.81 (0.79–4.13)
Muscle mass										
Normal	1		1		1		1		1	
Abnormal	1.10 (0.41–2.98)		1.28 (0.76–2.18)		3.89 (0.64–23.66)		1.44 (0.84–2.48)		0.96 (0.45–2.05)	
Proteinuria										
Negative	1		1		1		1		1	
Positive	3.40 (1.7–6.78)	3.71 (1.75–7.88)	1.44 (0.96–2.15)		1.22 (0.11–8.69)		1.49 (0.98–2.27)		1.12 (0.64–1.95)	
Albumin creatinine ratio										
< 30	1		1							
>=30	1.74 (0.69–4.4)		0.76 (0.43–1.34)		1.67 (0.19–14.6)		0.89 (0.47–1.71)			
On Tenofovir based regimen										
No	1		1		1		1		1	
Yes	1.45 (0.62–3.41)		0.97 (0.64–1.46)		1.72 (0.19–14.88)		3.22 (1.85–5.61)	1.87 (0.94–3.73)	0.63 (0.36–1.08)	
Duration on ART in years										
< 5	1		1		1		1		1	
6 to 10	0.49 (0.17–1.43)		0.79 (0.46–1.39)		0.75 (-,-)		0.92 (0.49–1.71)		0.78 (0.37–1.61)	
> 10	1.11 (0.43–2.89)		1.12 (0.64–1.98)		1.27 (-,-)		1.31 (0.71–2.42)		0.62 (0.11–0.39)	

OR Odds ratio ART Anti-retroviral therapy. *N* Number, eGFR estimated glomerular filtration rate* Adjusted for age, sex, blood pressure, viral suppression proteinuria, baseline CD4 T cell count

Results were similar when using CKD defined by eGFR < 60 ml/min/1.73m^2^ (Supplementary Table 3).

## Discussion

This is the first study to compare the prevalence and factors associated with CKD diagnosed by creatinine and cystatin C among YPLHIV in Uganda according to standard guidelines. We found highly variable prevalence depending on the definition, the estimating equation and the biomarker used. This was compounded by the commonly used GFR estimating equations being recommended for adults (CKDEPI 2021) or children (Schwartz) only, despite the highly variable physical and sexual maturity within this important age group where long-term disease management is critical. Using cystatin C eGFR measures consistently gave a substantially higher prevalence of CKD: using the Schwartz cystatin equation approximately 60% of YPLHIV had eGFR < 90mls/min/1.73m^2^. While dipstick proteinuria is anticipated in this population largely treated with anti-retroviral drugs, 10% of participants had substantially elevated levels of albuminuria. However, when participants with baseline abnormalities were remeasured at three months according to the gold-standard definition, overall prevalence of CKD was much lower. This might have been due to transient derangements in kidney functions caused by dehydration, physical stress, fever or acute kidney diseases that had resolved by the three-month follow-up visit [40, 41].

The highest prevalence (59%) using an eGFR cutoff < 90 ml/min/1.73m^2^ and the Schwartz cystatin C equation, at baseline which fell to 23% at three months follow-up, was very high. This is similar to a study done in 96 Nigerian YPLHIV aged 15 to 29 years which found 53.3% prevalence [42] and a Tanzanian study among 240 YPLHIV aged less than 14 years that showed a prevalence of 28% [43]. When kidney function was determined by eGFR below 60 ml/min/1.73m^2^ on two separate occasions at least three months apart, the prevalence of CKD was 0.8%. This is lower than in a study done in Zambia among children living with HIV aged 1 to 18 years that found a prevalence of 3.8% after 3 months [44]. However, the children in this Zambian study were younger than the ones in our study.

Using a eGFR cut off of less than 60 ml/min/1.73m^2^ excludes a large proportion of YPLHIV who are already showing signs of impaired kidney function such as an ACR > = 30 mg/g, proteinuria and hypertension, and who would benefit from early intervention to halt progression of their kidney disease [45]. Using this KDIGO recommended GFR cut off that was based on adult kidney function and markers can lead to underdiagnosis of CKD in children. Pottel et al. have shown that clinical manifestations of decreased kidney function in young people start at GFR less than 75 ml/min/1.73m^2^; they recommend that the CKD definition should be revised to reflect this [46]. This could be even higher for those with chronic conditions such as HIV. The recommendations should consider them as well.

KDIGO recommends that screening and surveillance for CKD be tailored to the specific high risk group [47]. Our study suggests that using sequential estimation of eGFR over three months excludes YPLHIV at risk of CKD, and might be misleading to the public health response whose goal is to halt progression and to predict those who are in danger of kidney failure or development of cardiovascular complications [47]. KDIGO further recommends that screening frequency should be based on the risk profile of the individual and potential to progress [48]. YPLHIV have the potential to progress due to the continued insult to the kidney, one abnormal eGFR measurement that shows reduced kidney function should be sufficient for them to be followed up regularly and managed.

Estimating GFR in our population was challenging as the different estimating equations and biomarkers gave very different results. This was worse as one transitioned from equations meant for those below 18 years to those equations meant for adults above 18 years. The difference in the eGFR was wide even in the same individual. It is difficult to determine the true estimate for CKD among YPLHIV using these estimating equations yet knowing the true estimate is important to plan the public health response for CKD [7]. Clinicians who seek to diagnose CKD and plan management may get confused about the true CKD status of an individual. Misdiagnosis and classification of YPLHIV removes the opportunity to intervene early to halt progression to kidney failure [49]. The KDIGO recommended CKDEPI equation was developed from Caucasians with a mean age of 47 years and might not be appropriate for younger adults [50]. It is not surprising that each of the estimating equations gave a different prevalence since each estimating equation reflects the characteristics of the population/dataset that was used to develop it [51]. There is an urgent need to develop estimating equations for Africans living in Africa.

The use of GFR alone doesn’t predict progression or mortality risk and other markers of kidney damage such as albuminuria or proteinuria are used [26, 52]. This has also been shown in a meta-analysis with over 27 million adult participants that showed lower levels of GFR and high levels of ACR were associated with more adverse outcomes such as kidney failure, cardiovascular events and hospitalisations [53]. When ACR was used, the prevalence was 10.1%. This is lower than that reported among a Tanzanian cohort of YPLHIV aged 1 to 14 years which found a prevalence of 20.1% [43]. However, the ACR was determined at a single time point and included younger children. Proteinuria prevalence was 29% which was high in such a young population. Proteinuria is an early marker of HIV associated nephropathy [54] and if persistent, is predictive of CKD status in children [55]. However, we measured proteinuria only at baseline and yet two positive out of three readings are used to diagnose persistent proteinuria [47].

Cystatin C emerged as a better biomarker than serum creatinine as eGFR calculated from Cystatin C was above CKD stage 1 more consistently for all those that had an increased ACR, proteinuria or hypertension which are markers of abnormal kidney function [12]. In this study, 12.4% of the participants had a BMI < 18.5 kg/m^2^, the use of serum creatinine to estimate GFR in this group would lead to underdiagnosis of CKD given the dependence of creatinine on nutritional status or muscle mass. Cystatin C was recommended by a recent study in three countries (Uganda, Malawi, and South Africa) as the better biomarker in Africans [4]. Cystatin C is not as widely available in Uganda and is more expensive than serum creatinine making it harder to access [8]. Cystatin C should be recommended for the diagnosis of CKD in YPLHIV as well.

The associated factors varied by equation and biomarker used. We found that age, sex, and HIV viral non-suppression were associated with CKD for most equations and that proteinuria, CD4 cell count, blood pressure, and being on a TDF regimen were not associated for all of them. Age was not associated with CKD for the cystatin C-based equations. A study among perinatally infected YPLHIV in South Africa with a mean age of 12.0 years found sex, but not age or blood pressure were associated with CKD [56]. Males were also found to have more CKD than females in a study in Zimbabwe [57]. TDF use was also not associated with CKD status in a cohort of American children with CKD [58].

One of the strengths of this study is that we estimated the eGFR and ACR at two different time points more than three months apart as recommended by KDIGO and were able to ascertain those that actually had CKD according to the standard definition of CKD. However, most of the GFR estimating equations and normal serum creatinine have not been validated in YPLHIV in resource-limited settings especially in Africa and this makes it that much harder to determine the abnormal values in YPLHIV [4, 59]. This could explain the low correlation between eGFR and the markers of kidney damage found in this study. We determined both markers of kidney damage (albuminuria and proteinuria) and function and could tell YPLHIV that were at risk of CKD progression. The biggest limitation is that we did not measure the GFR using either ioxehol or the nuclear tracers 99mTc-diethylenetriaminepentaacetic acid (DTPA) or ^51^Cr-EDTA [10] and so we are unable to tell how accurate the eGFR was. This study was carried out in an urban setting and the prevalence might be different in the rural parts of Uganda.

## Conclusion

CKD prevalence among YPLHIV in Uganda was much lower using serum creatinine-based estimates compared to cystatin C based estimates. Prevalence of kidney impairment was much higher at baseline compared to three months later. Cystatin C diagnosed more people with both kidney impairment and CKD but is more expensive in Uganda. The substantial prevalence of albuminuria and reduced eGFR suggest that HIV programs should prioritize screening for CKD among YPLHIV. The definition of CKD and best biomarker to use in YPLHIV should be further investigated to optimise detection of those with early abnormalities of kidney function. Estimating equations should be validated against measured GFR in young people to define how best to estimate GFR across older children and young adults in Africa.

## Electronic supplementary material

Below is the link to the electronic supplementary material.

Supplementary Material 1

## Data Availability

The data supporting the findings of this study are openly available in repository https://datacompass.lshtm.ac.uk/.

## Abbreviations

ACR: Albumin Creatinine Ratio
AIDS: Acquired Immune Deficiency Syndrome
ART: Anti-Retroviral Therapy
BIS: Bioimpedance Spectroscopy
BMI: Body Mass Index
CALHIV: Children and Adolescents living with HIV
CD4: Cluster of differentiation 4
CKD: Chronic Kidney Disease
CKD-EPI: Chronic Kidney Disease Epidemiology Collaboration
DM: Diabetes Mellitus
FAS: Full Age Spectrum
GFR: Glomerular Filtration Rate
HB: Haemoglobin
HIV: Human Immunodeficiency Virus
HIVAN: HIV associated Nephropathy
HW: Health Worker
IQR: Interquartile Range
KDIGO: Kidney Disease Improving Global Outcomes
KRT: Kidney replacement therapy
NCD’s: Non-Communicable Diseases
PLHIV: People Living with HIV AIDS
SSA: Sub Saharan Africa
USA: United States of America
WHO: World Health Organization
YPLHIV: Young People Living with HIV

## References

[1] Kovesdy CP. Epidemiology of chronic kidney disease: an update 2022. Kidney Int Supplements. 2022;12(1):7–11. 10.1016/j.kisu.2021.11.003 PMC9073222 35529086

[2] National Kidney Foundation. K/DOQI clinical practice guidelines for chronic kidney disease: evaluation, classification, and stratification. Am J Kidney Disease. 2002;39:S1–266. 11904577

[3] Bikbov B, Purcell CA, Levey AS, Smith M, Abdoli A, Abebe M, et al. Global, regional, and national burden of chronic kidney disease, 1990–2017: a systematic analysis for the global burden of Disease Study 2017. Lancet. 2020;395(10225):709–33. 32061315 10.1016/S0140-6736(20)30045-3 PMC7049905

[4] Fabian J, Kalyesubula R, Mkandawire J, Hansen CH, Nitsch D, Musenge E, et al. Measurement of kidney function in Malawi, South Africa, and Uganda: a multicentre cohort study. Lancet Global Health. 2022;10(8):e1159–69. 35839814 10.1016/S2214-109X(22)00239-X

[5] Fabian J, George JA, Etheredge HR, van Deventer M, Kalyesubula R, Wade AN, et al. Methods and reporting of kidney function: a systematic review of studies from Sub-saharan Africa. Clin Kidney J. 2019;12(6):778–87. 31807291 10.1093/ckj/sfz089 PMC6885675

[6] Naicker S, Fabian J. Risk factors for the development of chronic kidney disease with HIV/AIDS. Clin Nephrol. 2010;74(Suppl 1):S51–6. 20979964 10.5414/cnp74s051

[7] Stanifer JW, Muiru A, Jafar TH, Patel UD. Chronic kidney disease in low-and middle-income countries. Nephrol Dialysis Transplantation. 2016;31(6):868–74. 10.1093/ndt/gfv466 PMC4876969 27217391

[8] Kalyesubula R, Makanga G, Gyagenda JO, Atuhe D, Kansiime G, Kiggundu D et al. Nephrology in Uganda. Nephrol Worldw. 2021:75–83.

[9] George C, Mogueo A, Okpechi I, Echouffo-Tcheugui JB, Kengne AP. Chronic kidney disease in low-income to middle-income countries: the case for increased screening. BMJ Glob Health. 2017;2(2):e000256. 29081996 10.1136/bmjgh-2016-000256 PMC5584488

[10] Matsushita K, van der Velde M, Astor BC, Woodward M, Levey AS, de Jong PE, et al. Association of estimated glomerular filtration rate and albuminuria with all-cause and cardiovascular mortality in general population cohorts: a collaborative meta-analysis. Lancet. 2010;375(9731):2073–81. 20483451 10.1016/S0140-6736(10)60674-5 PMC3993088

[11] van der Velde M, Matsushita K, Coresh J, Astor BC, Woodward M, Levey A, et al. Lower estimated glomerular filtration rate and higher albuminuria are associated with all-cause and cardiovascular mortality. A collaborative meta-analysis of high-risk population cohorts. Kidney Int. 2011;79(12):1341–52. 21307840 10.1038/ki.2010.536

[12] Lopez-Giacoman S, Madero M. Biomarkers in chronic kidney disease, from kidney function to kidney damage. World J Nephrol. 2015;4(1):57. 25664247 10.5527/wjn.v4.i1.57 PMC4317628

[13] Levey AS, Titan SM, Powe NR, Coresh J, Inker LA. Kidney disease, race, and GFR Estimation. Clin J Am Soc Nephrol. 2020;15(8):1203–12. 32393465 10.2215/CJN.12791019 PMC7409747

[14] Inker LA, Eneanya ND, Coresh J, Tighiouart H, Wang D, Sang Y, et al. New Creatinine- and cystatin C–Based equations to Estimate GFR without Race. N Engl J Med. 2021;385(19):1737–49. 34554658 10.1056/NEJMoa2102953 PMC8822996

[15] Kashani K, Rosner MH, Ostermann M, Creatinine. From physiology to clinical application. Eur J Intern Med. 2020;72:9–14. 31708357 10.1016/j.ejim.2019.10.025

[16] Shlipak MG, Mattes MD, Peralta CA. Update on cystatin C: incorporation into clinical practice. Am J Kidney Dis. 2013;62(3):595–603. 23701892 10.1053/j.ajkd.2013.03.027 PMC3755100

[17] Seape T, Gounden V, van Deventer HE, Candy GP, George JA. Cystatin C- and creatinine-based equations in the assessment of renal function in HIV-positive patients prior to commencing highly active antiretroviral therapy. Ann Clin Biochem. 2016;53(1):58–66. 25766385 10.1177/0004563215579695

[18] Mauss S, Berger F, Kuschak D, Henke J, Hegener P, Wolf E, et al. Cystatin C as a marker of renal function is affected by HIV replication leading to an underestimation of kidney function in HIV patients. London, England: SAGE Publications Sage UK; 2008. 19195336

[19] Mian AN, Schwartz GJ. Measurement and estimation of glomerular filtration rate in children. Adv Chronic Kidney Dis. 2017;24(6):348–56. 29229165 10.1053/j.ackd.2017.09.011 PMC6198668

[20] Frigati LJ, Ameyan W, Cotton MF, Gregson CL, Hoare J, Jao J, et al. Chronic comorbidities in children and adolescents with perinatally acquired HIV infection in sub-saharan Africa in the era of antiretroviral therapy. Lancet Child Adolesc Health. 2020;4(9):688–98. 32359507 10.1016/S2352-4642(20)30037-7

[21] Jotwani V, Atta MG, Estrella MM. Kidney disease in HIV: moving beyond HIV-Associated Nephropathy. J Am Soc Nephrol. 2017;28(11):3142–54. 28784698 10.1681/ASN.2017040468 PMC5661296

[22] Kalyesubula R, Wearne N, Semitala FC, Bowa K. HIV-associated renal and genitourinary comorbidities in Africa. J Acquir Immune Defic Syndr. 2014;67(Suppl 1):S68–78. 25117962 10.1097/QAI.0000000000000259

[23] Purswani MU, Chernoff MC, Mitchell CD, Seage GR, Zilleruelo G, Abitbol C, et al. Chronic kidney disease associated with perinatal HIV infection in children and adolescents. Pediatr Nephrol. 2012;27(6):981–9. 22366874 10.1007/s00467-011-2097-1 PMC3715373

[24] Bhimma R, Purswani MU, Kala U. Kidney disease in children and adolescents with perinatal HIV-1 infection. J Int AIDS Soc. 2013;16(1):18596. 23782479 10.7448/IAS.16.1.18596 PMC3687339

[25] Innes S, Patel K. Non-communicable diseases in adolescents with perinatally-acquired HIV-1 infection in high-and low-income settings. Curr Opin HIV AIDS. 2018;13(3):187. 29432231 10.1097/COH.0000000000000458 PMC5934760

[26] Bunupuradah T, Phupitakphol T, Sophonphan J, Prasitsuebsai W, Anugulruengkitt S, Jantarabenjakul W, et al. Prevalence of persistent renal dysfunction in perinatally HIV-infected Thai adolescents. Pediatr Infect Dis J. 2018;37(1):66–70. 28719505 10.1097/INF.0000000000001684

[27] Bernheimer JM, Patten G, Makeleni T, Mantangana N, Dumile N, Goemaere E, et al. Paediatric HIV treatment failure: a silent epidemic. J Int AIDS Soc. 2015;18:20090. 26208630 10.7448/IAS.18.1.20090 PMC4514899

[28] Queremel Milani DA, Jialal I, Urinalysis. StatPearls Publishing, Treasure Island (FL); 2020 2020. 32491617

[29] Levin A, Stevens PE, Bilous RW, Coresh J, De Francisco AL, De Jong PE, et al. Kidney disease: improving global outcomes (KDIGO) CKD Work Group. KDIGO 2012 clinical practice guideline for the evaluation and management of chronic kidney disease. Kidney Int Supplements. 2013;3(1):1–150.

[30] Levey AS, Eckardt K-U, Dorman NM, Christiansen SL, Cheung M, Jadoul M et al. Nomenclature for kidney function and disease: executive summary and glossary from a Kidney Disease: Improving Global Outcomes (KDIGO) consensus conference. Kidney Diseases. 2020;6(5):309 – 17. 10.1159/000509359 PMC7745658 33490111

[31] Levey AS, Coresh J. Chronic kidney disease. Lancet. 2012;379(9811):165–80. 21840587 10.1016/S0140-6736(11)60178-5

[32] Schwartz GJ, Munoz A, Schneider MF, Mak RH, Kaskel F, Warady BA, et al. New equations to estimate GFR in children with CKD. J Am Soc Nephrology: JASN. 2009;20(3):629. 10.1681/ASN.2008030287 PMC2653687 19158356

[33] Schwartz GJ, Schneider MF, Maier PS, Moxey-Mims M, Dharnidharka VR, Warady BA, et al. Improved equations estimating GFR in children with chronic kidney disease using an immunonephelometric determination of cystatin C. Kidney Int. 2012;82(4):445–53. 22622496 10.1038/ki.2012.169 PMC3433576

[34] Inker LA, Schmid CH, Tighiouart H, Eckfeldt JH, Feldman HI, Greene T, et al. Estimating glomerular filtration rate from serum creatinine and cystatin C. N Engl J Med. 2012;367(1):20–9. 22762315 10.1056/NEJMoa1114248 PMC4398023

[35] Pottel H, Hoste L, Dubourg L, Ebert N, Schaeffner E, Eriksen BO, et al. An estimated glomerular filtration rate equation for the full age spectrum. Nephrol Dialysis Transplantation. 2016;31(5):798–806. 10.1093/ndt/gfv454 PMC4848755 26932693

[36] Björk J, Nyman U, Larsson A, Delanaye P, Pottel H. Estimation of the glomerular filtration rate in children and young adults by means of the CKD-EPI equation with age-adjusted creatinine values. Kidney Int. 2021;99(4):940–7. 33157151 10.1016/j.kint.2020.10.017

[37] Pierce CB, Muñoz A, Ng DK, Warady BA, Furth SL, Schwartz GJ. Age-and sex-dependent clinical equations to estimate glomerular filtration rates in children and young adults with chronic kidney disease. Kidney Int. 2021;99(4):948–56. 33301749 10.1016/j.kint.2020.10.047 PMC9083470

[38] Flynn JT, Falkner BE. New Clinical Practice Guideline for the management of high blood pressure in children and adolescents. Hypertens (Dallas Tex: 1979). 2017;70(4):683–6. 10.1161/HYPERTENSIONAHA.117.10050 28827475

[39] Thompson CG, Kim RS, Aloe AM, Becker BJ. Extracting the variance inflation factor and other multicollinearity diagnostics from typical regression results. Basic Appl Soc Psychol. 2017;39(2):81–90.

[40] Bongers C, Alsady M, Nijenhuis T, Tulp ADM, Eijsvogels TMH, Deen PMT, et al. Impact of acute versus prolonged exercise and dehydration on kidney function and injury. Physiol Rep. 2018;6(11):e13734. 29890037 10.14814/phy2.13734 PMC5995308

[41] Lameire NH, Levin A, Kellum JA, Cheung M, Jadoul M, Winkelmayer WC et al. Harmonizing acute and chronic kidney disease definition and classification: report of a Kidney Disease: Improving Global Outcomes (KDIGO) Consensus Conference. Kidney international. 2021;100(3):516 – 26. 10.1016/j.kint.2021.06.028 34252450

[42] Okafor UH, Unuigbe EI, Chukwuonye E. Prevalence and clinical and laboratory characteristics of kidney disease in anti-retroviral-naive human immunodeficiency virus-infected patients in South-South Nigeria. Saudi journal of kidney diseases and transplantation: an official publication of the Saudi Center for Organ Transplantation. Saudi Arabia. 2016;27(1):129–34. 10.4103/1319-2442.174155 26787579

[43] Fredrick F, Francis JM, Ruggajo PJ, Maro EE. Renal abnormalities among HIV infected children at Muhimbili National Hospital (MNH)-Dar es Salaam, Tanzania. BMC Nephrol. 2016;17:30. 27000018 10.1186/s12882-016-0242-6 PMC4800772

[44] Zimba KM. Prevalence and factors associated with renal dysfunction in HIV positive paediatric patients on highly active antiretroviral therapy at the paediatric centre of excellence of the university teaching hospital, in Lusaka, Zambia. Pediatr Nephrol. 2016;31(10):1778.

[45] Uhlig K, Johnson CA, Levey AS. New guidelines spell out approach to chronic kidney disease: early diagnosis of chronic kidney disease widens the window of opportunity to put effective treatments to work. Recent National Kidney Foundation clinical practice guidelines provide a simple definition, a practical staging system, and a stage-appropriate action plan. Patient Care. 2003;37(7):38–45.

[46] Pottel H, Hoste L, Delanaye P. Abnormal glomerular filtration rate in children, adolescents and young adults starts below 75 mL/min/1.73 m 2. Pediatr Nephrol. 2015;30:821–8. 25403744 10.1007/s00467-014-3002-5

[47] Levey A, Atkins R, Coresh J, Cohen E, Collins A, Eckardt K-U, et al. Chronic kidney disease as a global public health problem: approaches and initiatives–a position statement from kidney disease improving global outcomes. Kidney Int. 2007;72(3):247–59. 17568785 10.1038/sj.ki.5002343

[48] Shlipak MG, Tummalapalli SL, Boulware LE, Grams ME, Ix JH, Jha V, et al. The case for early identification and intervention of chronic kidney disease: conclusions from a kidney disease: improving global outcomes (KDIGO) Controversies Conference. Kidney Int. 2021;99(1):34–47. 10.1016/j.kint.2020.10.012 33127436

[49] Bhimma R. HIV-Related kidney diseases. In: Bobat R, editor. HIV infection in children and adolescents. Cham: Springer International Publishing; 2020. pp. 143–52.

[50] Levey AS, Stevens LA, Schmid CH, Zhang YL, Castro AF 3rd, Feldman HI, et al. A new equation to estimate glomerular filtration rate. Ann Intern Med. 2009;150(9):604–12. 19414839 10.7326/0003-4819-150-9-200905050-00006 PMC2763564

[51] Miller WG. Perspective on new equations for estimating glomerular filtration rate. Clin Chem. 2021;67(6):820–2. 33720356 10.1093/clinchem/hvab029

[52] Bökenkamp A. Proteinuria—take a closer look! Pediatr Nephrol. 2020;35(4):533–41. 31925536 10.1007/s00467-019-04454-w PMC7056687

[53] Consortium WGftCP. Estimated glomerular filtration rate, Albuminuria, and adverse outcomes: an individual-participant data Meta-Analysis. JAMA. 2023;330(13):1266–77. 37787795 10.1001/jama.2023.17002 PMC10548311

[54] Palau L, Menez S, Rodriguez-Sanchez J, Novick T, Delsante M, McMahon BA, et al. HIV-associated nephropathy: links, risks and management. HIV AIDS (Auckl). 2018;10:73–81. 29872351 10.2147/HIV.S141978 PMC5975615

[55] Beng H, Rakhmanina N, Moudgil A, Tuchman S, Ahn S-Y, Griffith C, et al. HIV-associated CKDs in children and adolescents. Kidney Int Rep. 2020;5(12):2292–300. 33305123 10.1016/j.ekir.2020.09.001 PMC7710839

[56] Frigati L, Mahtab S, Nourse P, Ray P, Perrazzo S, Machemedze T, et al. Prevalence of risk factors for chronic kidney disease in South African youth with perinatally acquired HIV. Pediatric nephrology (Berlin. Germany). 2018;34(2):313–8. 10.1007/s00467-018-4080-6 PMC6529608 30219929

[57] Byers BW, Drak D, Shamu T, Chimbetete C, Dahwa R, Gracey DM. Assessing renal impairment in treatment-naïve adolescents living with HIV commencing antiretroviral therapy in Zimbabwe. AIDS. 2023;37(5):789–94. 36728249 10.1097/QAD.0000000000003482

[58] Purswani M, Patel K, Kopp JB, Seage GR 3rd, Chernoff MC, Hazra R, et al. Tenofovir treatment duration predicts proteinuria in a multiethnic United States Cohort of children and adolescents with perinatal HIV-1 infection. Pediatr Infect Dis J. 2013;32(5):495–500. 23249917 10.1097/INF.0b013e31827f4eff PMC3800277

[59] Swanepoel CR, Atta MG, D’Agati VD, Estrella MM, Fogo AB, Naicker S, et al. Kidney disease in the setting of HIV infection: conclusions from a kidney disease: improving global outcomes (KDIGO) Controversies Conference. Kidney Int. 2018;93(3):545–59. 10.1016/j.kint.2017.11.007 PMC5983378 29398134

