# Supplementary material for: Comparison of the prevalence and associated factors of chronic kidney disease diagnosed by serum creatinine or cystatin C among young people living with HIV in Uganda
Source: BMC Nephrol. 2024 Nov 25;25:422. doi: 10.1186/s12882-024-03865-8 (PMC11590532; doi:10.1186/s12882-024-03865-8)
Supplement: Supplementary file 1 — Supplementary Material 1 [file 12882_2024_3865_MOESM1_ESM.docx]

**Supplementary Materials**

**Supplemental table 1 Difference in mean serum creatinine and cystatin C by selected variables**

| Variable | Serum creatinine |  | Serum Cystatin C |  |
| --- | --- | --- | --- | --- |
| Total (mean, SD) | 0.63 (0.15) |  | 0.81 (0.13) |  |
| **Sex** |  |  |  |  |
| Male | 0.70 (0.17) | <0.001 | 0.86 (0.13) | <0.001 |
| Female | 0.62 (0.12) |  | 0.78 (0.12) |  |
| **Age** |  |  |  |  |
| Children | 0.60 (0.13) | <0.001 | 0.81 (0.12) | 0.37 |
| Adults | 0.78 (0.15) |  | 0.81 (0.14) |  |
| **Viral suppression** |  |  |  |  |
| Suppressed | 0.66 (0.15) | 0.02 | 0.81 (0.13) | 0.99 |
| Non-suppressed | 0.61 (0.14) |  | 0.86 (0.14) |  |
| **Stunting** |  |  |  |  |
| Not stunted | 0.63 (0.12) | 0.0002 | 0.81 (0.12) | 0.75 |
| Stunted | 0.57 (0.13) |  | 0.82 (0.13) |  |
| **BMI** |  |  |  |  |
| Normal/High | 0.66 (0.15) | 0.08 | 0.81 (0.13) | 0.94 |
| Low | 0.63 (0.14) |  | 0.84 (0.12) |  |
| Proteinuria |  |  |  |  |
| Negative | 0.65 (0.15) |  | 0.80 (0.12) | 0.98 |
| Positive | 0.67 (0.16) | 0.84 | 0.83 (0.14) |  |
| Albumin creatinine ratio |  |  |  |  |
| <=30 | 0.66 (0.15) |  | 0.81 (0.13) |  |
| >30 | 0.65 (0.16) |  | 0.81 (0.15) |  |

**Supplementary table 2. CKD prevalence according to different estimating equations**

|  | Adults (18 -24 years) n=166 | | | Children (10-17 years) n=335 | | | All ages (10-24 years) n=495 | | |
| --- | --- | --- | --- | --- | --- | --- | --- | --- | --- |
| Creatinine | Equation | 60-90 | <60 | Equation | 60-90 | <60 | Equation | 60-90 | <60 |
|  | CKDEPI 2009 | 7 (4.3) | 0 (0) | Bedside Schwartz | 53 (15.8) | 0 (0) | FAScr | 134 (27.0) | 2 (0.4) |
|  | CKDEPI 2021 | 5 (3.0) | 0 (0) | Lund Malmo18 | 26 (7.8) | 0 (0) | CKDEPI40 | 83 (16.8) | 0 (0) |
|  | MDRD | 18 (11.0) | 0 (0) | Schwartz Lyon | 198 (59.8) | 8 (2.4) | EKFC | 37 (7.5) | 0 (0) |
|  | Cockroft Gault | 24 (14.7) | 0 (0) | BCCH2 | 5 (1.5) | 0 (0) | U25 | 127 (25.7) | 0 (0) |
| Cystatin C | CKDEPI 2012 | 18 (11) | 1 (0.3) | Schwartz# | 191 (57.7) | 5 (1.5) | Grubb | 51 (10.3) | 1 (0.2) |
|  | Hojs | 15 (9.1) | 0 (0) | CAPA | 19 (5.7) | 0 (0) | U25 cyst C | 174 (35.2) | 2 (0.2) |
|  | Simple | 3 (1.8) | 0 (0) | Larsson | 88 (26.6) | 4 (1.2) | FAS cyst C | 46 (9.3) | 0 (0) |
| Creatinine and Cystatin C | CKDEPI 2012 | 6 (3.6) | 0 (0) | Zapitelli | 41 (12.4) | 1 (0.3) | FAS combi | 29 (5.9) | 0 (0) |
|  | CKDEPI 2021 | 5 (3.0) | 0(0) | Bouvet | 100 (30.2) | 0 (0) | U25 combi | 113 (22.9) | 0 (0) |

**Supplementary table 3 Factors associated with having CKD according to eGFR less than 60ml/min/1.73m^2^**

|  | **Schwartz Cystatin C** | | **Bedside Schwartz** | |
| --- | --- | --- | --- | --- |
|  | **Unadjusted OR** | **Adjusted OR** | **Unadjusted OR** | **Adjusted OR** |
| **Age in years** | 1.01 (0.82-1.24) | 1.01 (0.82-1.23) | 1.76 (1.22-2.86) | 1.72 (1.18-2.86) |
| **Age categorized** |  |  |  |  |
| >18 | 1 |  | 1 |  |
| <18 | 1.24 (0.20-13.21) |  | 0.09 (0-0.75) |  |
| **Sex** |  |  |  |  |
| Females | 1 |  | 1 |  |
| Males | 3.26 (0.53-34.57) | 3.28 (0.53-34.74) | 6.88 (0.85-'~) | 4.88 (0.64-~) |
| **Social Economic status** | |  |  |  |
| Least | 1 |  | 1 |  |
| Middle | 0.75 (0.62-6.64) |  | 1.12 (0-44.06) |  |
| Highest | 0.69 (0.06-6.15) |  | 3.17 (0.25-168.2) |  |
| **Body Mass Index** |  |  |  |  |
| Normal | 1 |  | 1 |  |
| Underweight | 1.53 (0.20-11.57) |  | 0.29 (0-2.30) |  |
| Overweight | 2.24 (0.63-2.43) |  | 1.25 (0-10.23) |  |
| **Weight in Kg** | 1.02 (0.96-1.07) |  | 1.04 (0.97-1.11) |  |
| **Stunting** |  |  |  |  |
| Not stunted | 1 |  | 1 |  |
| Stunted | 1.11 (0.02-11.47) |  | - |  |
| **Mid Upper Arm Circumference** | |  |  |  |
| Normal | 1 |  | 1 |  |
| Malnourished | 7.66 (1.09-46.92) |  | 1.84 (0-14.95) |  |
| **Muscle mass** |  |  |  |  |
| Normal | 1 |  | 1 |  |
| Abnormal | 0.95 (0.02-8.00) |  | 1.91 (0.04-24.16) |  |
| **Blood pressure** |  |  |  |  |
| Normal | 1 |  | 1 | 1 |
| Elevated | 0.66 (0-4.65) | 1.42 (0.79-2.59) | 1.41 (0-13.26) |  |
| Hypertensive | 0.73 (0.02-6.16) | 1.14 (0.67-1.94) | 1.47 (0.02-18.62) |  |
| **Proteinuria** |  |  |  |  |
| Negative | 1 |  | 1 |  |
| Positive | 1.85 (0.27-11.13) |  | 0.82 (0.02-10.28) |  |
| **Albumin creatinine ratio** | |  |  |  |
| <30 | 1 |  |  |  |
| >30 | 3.41 (0.31-21.46) |  | 2.79 (0.05-35.59) |  |
| **Currently on Tenofovir based regimen** | | |  |  |
| No | 1 |  | 1 |  |
| Yes | 0.85 (0.13-9.07) |  | 1.03 (0.08-54.36) |  |
| **Duration on ART in years** | |  |  |  |
| < 5 | 1 |  | 1 |  |
| 6 to 10 | 0.94 (0.07-50.03) |  | 0.31 (0.008-~) |  |
| >10 | 0.99 (0.78-52.75) |  | 1.28 (0.13-~) |  |
| **CD4 T cell count at baseline** | |  |  |  |
| >500 | 1 |  | 1 |  |
| 200-500 | 1.53 (0.13-10.87) |  | 6.15 (0.32-366.36) |  |
| <200 | 1.32 (0.02-13.68) |  | 5.31 (0.07-420) |  |
| **Viral suppression** |  |  |  |  |
| Suppressed | 1 | 1 | 1 |  |
| Non suppressed | 1.38 (0.03-11.75) | 2.29 (1.18-4.44) | 1.56 (0-12.67) |  |
